# 2-Methyl­sulfanyl-4-(3-pyrid­yl)pyrimidine

**DOI:** 10.1107/S1600536809037945

**Published:** 2009-09-26

**Authors:** Ren-Jun Du, Jian-Qiang Wang, Sheng Bi, Yi-Xin Zhou, Cheng Guo

**Affiliations:** a College of Science, Nanjing University of Technology, Xinmofan Road No. 5 Nanjing, Nanjing 210009, People’s Republic of China

## Abstract

In the title compound, C_10_H_9_N_3_S, the dihedral angle between the aromatic rings is 8.09 (14)°. In the crystal, a C—H⋯N interaction links the molecules, forming chains.

## Related literature

For bond-length data, see: Allen *et al.* (1987 ▶). For applications of pyrimidine derivatives, see: Mahboobi *et al.* (2008 ▶).
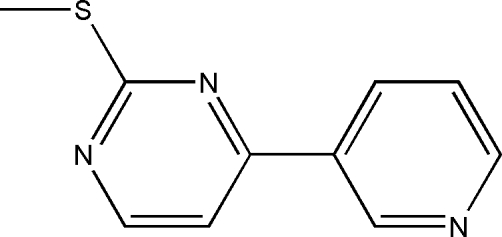


## Experimental

### 

#### Crystal data

C_10_H_9_N_3_S
*M* *_r_* = 203.26
Monoclinic, 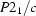

*a* = 4.0010 (8) Å
*b* = 13.713 (3) Å
*c* = 17.877 (4) Å
β = 96.35 (3)°
*V* = 974.8 (3) Å^3^
*Z* = 4
Mo *K*α radiation
μ = 0.29 mm^−1^
*T* = 293 K
0.30 × 0.10 × 0.10 mm

#### Data collection

Enraf–Nonius CAD-4 diffractometer
Absorption correction: ψ scan (Vorob’ev *et al*., 2006 ▶) *T*_min_ = 0.918, *T*_max_ = 0.971
2025 measured reflections
1758 independent reflections
1340 reflections with *I* > 2σ(*I*)
*R*_int_ = 0.025
3 standard reflections every 200 reflections intensity decay: 1%

#### Refinement

*R*[*F* ^2^ > 2σ(*F* ^2^)] = 0.051
*wR*(*F* ^2^) = 0.159
*S* = 1.02
1758 reflections
127 parameters
H-atom parameters constrained
Δρ_max_ = 0.26 e Å^−3^
Δρ_min_ = −0.19 e Å^−3^

### 

Data collection: *CAD-4 EXPRESS* (Enraf–Nonius, 1994 ▶); cell refinement: *CAD-4 EXPRESS*; data reduction: *XCAD4* (Harms & Wocadlo, 1995 ▶); program(s) used to solve structure: *SHELXS97* (Sheldrick, 2008 ▶); program(s) used to refine structure: *SHELXL97* (Sheldrick, 2008 ▶); molecular graphics: *SHELXL97*; software used to prepare material for publication: *SHELXL97*.

## Supplementary Material

Crystal structure: contains datablocks global, I. DOI: 10.1107/S1600536809037945/vm2004sup1.cif

Structure factors: contains datablocks I. DOI: 10.1107/S1600536809037945/vm2004Isup2.hkl

Additional supplementary materials:  crystallographic information; 3D view; checkCIF report

## Figures and Tables

**Table 1:** Hydrogen-bond geometry (Å, °)

*D*—H⋯*A*	*D*—H	H⋯*A*	*D*⋯*A*	*D*—H⋯*A*
C3—H3*A*⋯N3^i^	0.93	2.58	3.487 (4)	164
C10—H10*A*⋯N2	0.93	2.44	2.798 (4)	103

Symmetry code: (i) 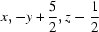.

**Fig. 1.**
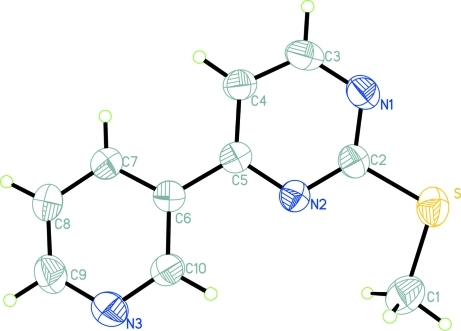
The molecular structure of (I), showing the atom-numbering scheme and displacement ellipsoids at the 30% probability level.

**Fig. 2.**
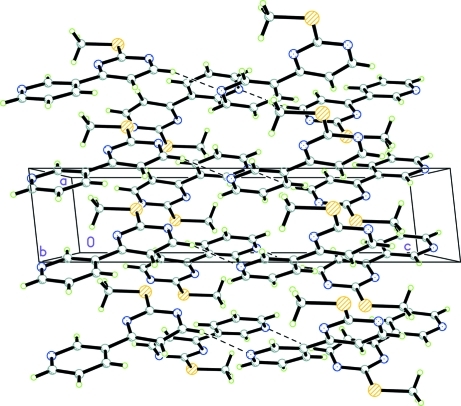
A packing diagram of (I). Possible intermolecular hydrogen bonds are shown as dashed lines.

## supplementary crystallographic information

### Comment

Some derivatives of pyrimidine are important chemical materials used as starting material for antineoplastic drugs (Mahboobi *et al.*, 2008). We report here the crystal structure of the title compound, (I). The molecular structure of (I) is shown in Fig. 1, and the selected geometric parameters are given in Table 1. The bond lengths and angles (Table 1) are within normal ranges (Allen *et al.*, 1987). A packing diagram of (I) is shown in Fig. 2.

### Experimental

To a mixture of 2-methyl-4-(pyridin-3-yl)pyrimidine hydrosulfide (20.0 g, 0.11 mol) and sodium hydride solution (1*M*, 106 ml), methyl iodide (15 g) was added slowly and was stirred for 2 h at 273 K. The reaction mixture was filtered, washed with water, and dried to give (I) (19.9 g). Pure compound (I) was obstained by crystallizing from ethanol solution. Crystals of (I) suitable for X-ray diffraction were obstained by slow evaporation of an cyclohexane solution.

### Refinement

All H atoms bonded to the C atoms were placed geometrically at the distances of 0.93–0.97 Å, and included in the refinement in riding motion approximation with *U*_iso_(H) = 1.2 or 1.5*U*_eq_ of the carrier atom.

### Crystal data

**Table:**

C_10_H_9_N_3_S	*F*(000) = 424
*M**_r_* = 203.26	*D*_x_ = 1.385 Mg m^−^^3^
Monoclinic, *P*2_1_/*c*	Mo *K*α radiation, λ = 0.71073 Å
Hall symbol: -P 2ybc	Cell parameters from 25 reflections
*a* = 4.0010 (8) Å	θ = 9–13°
*b* = 13.713 (3) Å	µ = 0.29 mm^−^^1^
*c* = 17.877 (4) Å	*T* = 293 K
β = 96.35 (3)°	Block, colorless
*V* = 974.8 (3) Å^3^	0.30 × 0.10 × 0.10 mm
*Z* = 4	

### Data collection

**Table:**

Enraf–Nonius CAD-4 diffractometer	1340 reflections with *I* > 2σ(*I*)
Radiation source: fine-focus sealed tube	*R*_int_ = 0.025
graphite	θ_max_ = 25.3°, θ_min_ = 1.9°
ω/2θ scans	*h* = 0→4
Absorption correction: ψ scan (Vorob'ev et al., 2006)	*k* = 0→16
*T*_min_ = 0.918, *T*_max_ = 0.971	*l* = −21→21
2025 measured reflections	3 standard reflections every 200 reflections
1758 independent reflections	intensity decay: 1%

### Refinement

**Table:**

Refinement on *F*^2^	Primary atom site location: structure-invariant direct methods
Least-squares matrix: full	Secondary atom site location: difference Fourier map
*R*[*F*^2^ > 2σ(*F*^2^)] = 0.051	Hydrogen site location: inferred from neighbouring sites
*wR*(*F*^2^) = 0.159	H-atom parameters constrained
*S* = 1.02	*w* = 1/[σ^2^(*F*_o_^2^) + (0.1*P*)^2^] where *P* = (*F*_o_^2^ + 2*F*_c_^2^)/3
1758 reflections	(Δ/σ)_max_ < 0.001
127 parameters	Δρ_max_ = 0.26 e Å^−^^3^
0 restraints	Δρ_min_ = −0.19 e Å^−^^3^

### Special details

**Table:**

Geometry. All e.s.d.'s (except the e.s.d. in the dihedral angle between two l.s. planes) are estimated using the full covariance matrix. The cell e.s.d.'s are taken into account individually in the estimation of e.s.d.'s in distances, angles and torsion angles; correlations between e.s.d.'s in cell parameters are only used when they are defined by crystal symmetry. An approximate (isotropic) treatment of cell e.s.d.'s is used for estimating e.s.d.'s involving l.s. planes.
Refinement. Refinement of *F*^2^ against ALL reflections. The weighted *R*-factor *wR* and goodness of fit *S* are based on *F*^2^, conventional *R*-factors *R* are based on *F*, with *F* set to zero for negative *F*^2^. The threshold expression of *F*^2^ > σ(*F*^2^) is used only for calculating *R*-factors(gt) *etc*. and is not relevant to the choice of reflections for refinement. *R*-factors based on *F*^2^ are statistically about twice as large as those based on *F*, and *R*- factors based on ALL data will be even larger.

### Fractional atomic coordinates and isotropic or equivalent isotropic displacement parameters (Å^2^)

**Table:**

	*x*	*y*	*z*	*U*_iso_*/*U*_eq_	
S	0.4309 (2)	0.94155 (5)	0.29123 (4)	0.0509 (3)	
N1	0.6539 (7)	1.07222 (17)	0.20518 (13)	0.0457 (6)	
C1	0.4170 (9)	0.9369 (2)	0.39062 (19)	0.0604 (9)	
H1B	0.3104	0.8775	0.4036	0.091*	
H1C	0.2913	0.9916	0.4060	0.091*	
H1D	0.6417	0.9392	0.4157	0.091*	
N2	0.7361 (5)	1.10742 (16)	0.33703 (12)	0.0360 (5)	
C2	0.6299 (7)	1.05331 (19)	0.27802 (15)	0.0378 (6)	
C3	0.8168 (8)	1.1546 (2)	0.19312 (16)	0.0477 (8)	
H3A	0.8455	1.1712	0.1438	0.057*	
N3	1.0852 (8)	1.2696 (2)	0.52338 (14)	0.0599 (8)	
C4	0.9438 (7)	1.2158 (2)	0.24957 (15)	0.0405 (7)	
H4A	1.0587	1.2722	0.2391	0.049*	
C5	0.8963 (6)	1.19124 (18)	0.32285 (14)	0.0342 (6)	
C6	1.0102 (7)	1.25258 (18)	0.38859 (15)	0.0362 (6)	
C7	1.1366 (8)	1.3459 (2)	0.38145 (16)	0.0475 (7)	
H7A	1.1536	1.3722	0.3341	0.057*	
C8	1.2363 (8)	1.3989 (2)	0.44521 (17)	0.0533 (8)	
H8A	1.3228	1.4615	0.4416	0.064*	
C9	1.2066 (8)	1.3584 (2)	0.51438 (17)	0.0534 (8)	
H9A	1.2750	1.3951	0.5571	0.064*	
C10	0.9898 (8)	1.2193 (2)	0.46139 (15)	0.0498 (8)	
H10A	0.9027	1.1572	0.4670	0.060*	

### Atomic displacement parameters (Å^2^)

**Table:**

	*U*^11^	*U*^22^	*U*^33^	*U*^12^	*U*^13^	*U*^23^
S	0.0491 (5)	0.0433 (5)	0.0608 (6)	−0.0072 (3)	0.0091 (4)	−0.0078 (4)
N1	0.0512 (15)	0.0455 (14)	0.0405 (14)	0.0041 (11)	0.0055 (11)	−0.0050 (11)
C1	0.061 (2)	0.055 (2)	0.066 (2)	−0.0093 (16)	0.0120 (17)	0.0099 (16)
N2	0.0350 (12)	0.0345 (12)	0.0389 (12)	0.0026 (10)	0.0056 (9)	−0.0009 (10)
C2	0.0348 (14)	0.0369 (14)	0.0419 (15)	0.0070 (12)	0.0049 (11)	−0.0032 (12)
C3	0.0560 (19)	0.0532 (18)	0.0355 (14)	0.0091 (15)	0.0124 (13)	0.0025 (13)
N3	0.085 (2)	0.0567 (16)	0.0380 (14)	−0.0123 (15)	0.0048 (13)	−0.0044 (12)
C4	0.0448 (17)	0.0391 (15)	0.0389 (14)	0.0016 (12)	0.0097 (12)	0.0023 (12)
C5	0.0302 (13)	0.0338 (13)	0.0388 (14)	0.0062 (11)	0.0044 (11)	0.0028 (11)
C6	0.0340 (14)	0.0365 (15)	0.0379 (14)	0.0039 (11)	0.0033 (11)	0.0013 (11)
C7	0.0530 (18)	0.0460 (17)	0.0428 (16)	−0.0105 (14)	0.0024 (13)	0.0053 (13)
C8	0.059 (2)	0.0459 (17)	0.0540 (18)	−0.0161 (15)	0.0022 (15)	−0.0027 (15)
C9	0.058 (2)	0.0532 (19)	0.0476 (18)	−0.0074 (16)	0.0010 (15)	−0.0114 (14)
C10	0.071 (2)	0.0396 (15)	0.0402 (16)	−0.0079 (15)	0.0102 (14)	0.0009 (13)

### Geometric parameters (Å, °)

**Table:**

S—C2	1.755 (3)	N3—C9	1.328 (4)
S—C1	1.785 (3)	C4—C5	1.386 (4)
N1—C3	1.333 (4)	C4—H4A	0.9300
N1—C2	1.342 (3)	C5—C6	1.476 (4)
C1—H1B	0.9600	C6—C7	1.387 (4)
C1—H1C	0.9600	C6—C10	1.390 (4)
C1—H1D	0.9600	C7—C8	1.374 (4)
N2—C2	1.321 (3)	C7—H7A	0.9300
N2—C5	1.354 (3)	C8—C9	1.373 (4)
C3—C4	1.367 (4)	C8—H8A	0.9300
C3—H3A	0.9300	C9—H9A	0.9300
N3—C10	1.325 (4)	C10—H10A	0.9300
			
C2—S—C1	103.26 (14)	N2—C5—C4	120.0 (2)
C3—N1—C2	114.2 (2)	N2—C5—C6	116.5 (2)
S—C1—H1B	109.5	C4—C5—C6	123.5 (2)
S—C1—H1C	109.5	C7—C6—C10	116.7 (3)
H1B—C1—H1C	109.5	C7—C6—C5	122.4 (2)
S—C1—H1D	109.5	C10—C6—C5	120.9 (2)
H1B—C1—H1D	109.5	C8—C7—C6	119.2 (3)
H1C—C1—H1D	109.5	C8—C7—H7A	120.4
C2—N2—C5	116.4 (2)	C6—C7—H7A	120.4
N2—C2—N1	128.0 (3)	C9—C8—C7	119.2 (3)
N2—C2—S	119.6 (2)	C9—C8—H8A	120.4
N1—C2—S	112.4 (2)	C7—C8—H8A	120.4
N1—C3—C4	123.3 (3)	N3—C9—C8	123.3 (3)
N1—C3—H3A	118.3	N3—C9—H9A	118.3
C4—C3—H3A	118.3	C8—C9—H9A	118.3
C10—N3—C9	116.8 (3)	N3—C10—C6	124.8 (3)
C3—C4—C5	118.1 (3)	N3—C10—H10A	117.6
C3—C4—H4A	121.0	C6—C10—H10A	117.6
C5—C4—H4A	121.0		
			
C5—N2—C2—N1	−1.7 (4)	N2—C5—C6—C7	−171.2 (3)
C5—N2—C2—S	178.06 (18)	C4—C5—C6—C7	8.4 (4)
C3—N1—C2—N2	2.6 (4)	N2—C5—C6—C10	7.7 (4)
C3—N1—C2—S	−177.23 (19)	C4—C5—C6—C10	−172.7 (3)
C1—S—C2—N2	2.4 (3)	C10—C6—C7—C8	0.7 (4)
C1—S—C2—N1	−177.8 (2)	C5—C6—C7—C8	179.7 (3)
C2—N1—C3—C4	−1.2 (4)	C6—C7—C8—C9	−0.3 (5)
N1—C3—C4—C5	−0.8 (4)	C10—N3—C9—C8	−0.1 (5)
C2—N2—C5—C4	−0.6 (4)	C7—C8—C9—N3	0.0 (5)
C2—N2—C5—C6	179.0 (2)	C9—N3—C10—C6	0.5 (5)
C3—C4—C5—N2	1.7 (4)	C7—C6—C10—N3	−0.8 (5)
C3—C4—C5—C6	−177.8 (2)	C5—C6—C10—N3	−179.8 (3)

### Hydrogen-bond geometry (Å, °)

**Table:**

*D*—H···*A*	*D*—H	H···*A*	*D*···*A*	*D*—H···*A*
C3—H3A···N3^i^	0.93	2.58	3.487 (4)	164
C10—H10A···N2	0.93	2.44	2.798 (4)	103

Symmetry codes: (i) *x*, −*y*+5/2, *z*−1/2.
